# Relationships between Cerebral Vasculopathies and Microinfarcts in a Community-Based Cohort of Older Adults

**DOI:** 10.3390/jcm12113807

**Published:** 2023-06-01

**Authors:** Mo-Kyung Sin, Yan Cheng, Jeffrey M. Roseman, Edward Zamrini, Ali Ahmed

**Affiliations:** 1 College of Nursing, Seattle University, Seattle, WA 98122, USA; 2 Biomedical Informatics Center, School of Medicine & Health Sciences, George Washington University, Washington, DC 20052, USA; yan_cheng@gwu.edu (Y.C.); ezamrini@irvineclinical.com (E.Z.); ali.ahmed@va.gov (A.A.); 3 Department of Epidemiology, University of Alabama at Birmingham, Birmingham, AL 35294, USA; bush@uab.edu; 4 Division of Neurology, Irvine Clinical Research, Irvine, CA 92614, USA; 5 Health and Aging, VA Medical Center, Washington, DC 20060, USA; 6 School of Medicine, Georgetown University, Washington, DC 20057, USA

**Keywords:** arteriolosclerosis, cerebral amyloid angiopathy, microinfarcts, cortical, subcortical

## Abstract

Cerebral microinfarcts are associated with cognitive impairment and dementia. Small vessel diseases such as cerebral arteriolosclerosis and cerebral amyloid angiography (CAA) have been found to be associated with microinfarcts. Less is known about the associations of these vasculopathies with the presence, numbers, and location of microinfarcts. These associations were examined in the clinical and autopsy data of 842 participants in the Adult Changes in Thought (ACT) study. Both vasculopathies were categorized by severity (none, mild, moderate, and severe) and region (cortical and subcortical). Odds ratios (OR) and 95% CIs for microinfarcts associated with arteriolosclerosis and CAA adjusted for possible modifying covariates such as age at death, sex, blood pressure, *APOE* genotype, Braak, and CERAD were estimated. 417 (49.5%) had microinfarcts (cortical, 301; subcortical, 249), 708 (84.1%) had cerebral arteriolosclerosis, 320 (38%) had CAA, and 284 (34%) had both. Ors (95% CI) for any microinfarct were 2.16 (1.46–3.18) and 4.63 (2.90–7.40) for those with moderate (*n* = 183) and severe (*n* = 124) arteriolosclerosis, respectively. Respective Ors (95% CI) for the number of microinfarcts were 2.25 (1.54–3.30) and 4.91 (3.18–7.60). Similar associations were observed for cortical and subcortical microinfarcts. Ors (95% Cis) for the number of microinfarcts associated with mild (*n* = 75), moderate (*n* = 73), and severe (*n* = 15) amyloid angiopathy were 0.95 (0.66–1.35), 1.04 (0.71–1.52), and 2.05 (0.94–4.45), respectively. Respective Ors (95% Cis) for cortical microinfarcts were 1.05 (0.71–1.56), 1.50 (0.99–2.27), and 1.69 (0.73–3.91). Respective Ors (95% Cis) for subcortical microinfarcts were 0.84 (0.55–1.28), 0.72 (0.46–1.14), and 0.92 (0.37–2.28). These findings suggest a significant association of cerebral arteriolosclerosis with the presence, number, and location (cortical and subcortical) of microinfarcts, and a weak and non-significant association of CAA with each microinfarct, highlighting the need for future research to better understand the role of small vessel diseases in the pathogenesis of cerebral microinfarcts.

## 1. Introduction

Cerebral microinfarcts play an important role in the clinical manifestation and severity of cognitive impairment and dementia [1,2,3]. While systemic risk factors of cerebral microinfarcts, such as hypertension and myocardial infarction, have been well studied [4,5,6], less is known about local risk factors, such as vasculopathies. Arteriolosclerosis and cerebral amyloid angiopathy (CAA) are the two most commonly found cerebral arteriopathies in older adults [2]. Arteriolosclerosis is a cerebral small vessel disease (thickening and hardening of the walls of the arteries in the brain) commonly found in subcortical regions [7] and leads to ischemic strokes, hemorrhagic strokes, or vascular dementia [8]. CAA, amyloid β protein contained in blood vessels within the brain, occurs primarily in the medium-sized arteries and arterioles in the leptomeningeal and cortical regions [9,10]. The presence of CAA can lead to hemorrhages ranging from micro hemorrhages to aneurysms, resulting in cognitive impairment and even death [11]. A higher severity of each of the cerebral vasculopathies has been reported to be associated with a greater burden of microinfarcts [12,13,14,15]. Clinical expression of dementia or cognitive impairment is associated with lesions in specific brain regions [16]. Despite evidence that cortical microinfarcts are specifically associated with poor global cognition, semantic memory, perceptual speed, and visuospatial abilities [17], the influence of vasculopathies on the location of microinfarcts remains understudied. Few studies have examined the relationships between vasculopathies and the number and location of microinfarcts. Thus, this study examined the relationships of cerebral vasculopathies (arteriolosclerosis, CAA) with the presence, numbers, and regions of microinfarcts. 

## 2. Materials and Methods

### 2.1. Data and Participants 

We used data from the Adult Changes in Thought (ACT) study. The ACT is a prospective cohort study that enrolled community-dwelling adults aged ≥ 65 years without dementia. Participants were members of Kaiser Permanente Washington (formerly Group Health). Total enrollment over the course of the study is >5000. ACT began in 1994–1996 with an enrollment of 2581 participants and an expansion cohort of 811 participants enrolled in 2000–2003 [18,19]. In 2004, the study began continuous enrollment to replace attrition from dementia, dropout, and death, ensuring a consistent cohort of ≥2000 at risk for dementia. Data were collected on demographics and clinical variables and from blood samples during enrollment and follow-up visits. Participants were also asked whether they would allow an autopsy on their brains after they die. As of March 2022, the ACT has 850 autopsies. The study participants underwent biennial cognitive screening, and those who screened “positive” (Cognitive Abilities Screening Instrument score ≤ 86) underwent a full neuropsychological evaluation. The study completeness of the follow-up index is >97% [20]. The current study is based on 842 ACT participants who had autopsy data on cerebral microinfarcts and cerebral vasculopathies. The study protocols were approved by the Kaiser Permanente Washington Institutional Review Boards. The Seattle University IRB has determined the study to be exempt from IRB review in accordance with federal regulation criteria.

### 2.2. Cerebral Microinfarcts 

Cerebral microinfarcts were identified during an autopsy by extensive neuropathological examinations performed by board-certified neuropathologists ensuring the data met current diagnostic criteria, including the National Institute on Aging–Alzheimer’s Association (NIA-AA) consensus guidelines [21,22]. A rapid autopsy dissection on cortical brain regions (frontal, temporal, parietal, and occipital) and subcortical regions (caudate nucleus, putamen, internal capsule, and thalamus) were obtained on all cases with post-mortem interval (PMI) < 8 h to ensure highest quality research tissue. All ACT brains underwent H&E/LFB staining for every sampled region as part of the standard diagnostic neurohistology workup [23]. Examiners were blinded to clinical and pathologic diagnoses. Cerebral microinfarcts were categorized into three levels: none, one, and multiple (≥2). Chronic microinfarcts in cortical gray matter regions (frontal, temporal, occipital, and parietal) were classified as cortical, and those with a caudate nucleus, putamen, internal capsule, and thalamus were classified as subcortical. 

### 2.3. Cerebral Vasculopathies 

Arteriolosclerosis was assessed by histological examination using existing H&E-stained sections from each region of interest. The severity of this pathology was graded based on the concentric hyaline thickening of vessel walls. Smaller arterioles, less than approximately 50 microns, were evaluated. The severity was categorized into four levels: none, mild (mild thickening of the vessel media, mild fibrosis), moderate (partial loss of smooth muscle cells in the media, moderate hyaline fibrosis), and severe (complete loss of smooth muscle cells in the media, severe hyaline fibrosis, lumen stenosis) [24,25].

Cerebral amyloid angiopathy (CAA) was assessed using Congo red histochemical stains to assess the degree of compacted amyloid β deposits in the brain leptomeninges and occipital cortex. Congo red stains amyloid protein aggregates when in a compacted form [13]. Congophilic amyloid in blood vessels is called CAA, and its severity is ranked on a four-point scale: none; mild, amyloid restricted to the tunica media without significant destruction of smooth muscle cells; moderate, the tunica media replaced by amyloid and thicker than normal; or severe, extensive amyloid deposition with focal wall fragmentation or even double barreling of the vessel wall, microaneurysm formation, fibrinoid necrosis, and leakage of blood through the blood vessel wall [26,27].

### 2.4. Other Variables

The covariates to be adjusted for included age at death, sex, last mean systolic and last mean diastolic blood pressures, antihypertensive medication use, APOE e4, Braak, and CERAD. They were selected in an effort to minimize their effects on the specific vasculopathy/microinfarcts relationships. The last mean blood pressure was defined as the mean of the last four consecutive measurements from ACT visits closest to death in this study. A study reported that a minimum of a 5-year time period is required to have progressive development of vascular impairment occur [28]. Data on the use of antihypertensive medications were collected from participants’ self-reports in the four consecutive visits. Antihypertensive medication use was assessed using the question, “Are you taking prescribed medication for blood pressure now?” and was categorized as “never used”, “current user”, and “past user” in four consecutive data points. In this study, if a patient was a “current user” during the last four visits, then the patient would be classified as ever-used antihypertensive medication. APOE genotyping has been completed by the University of Washington Alzheimer’s Disease Research Center Genotyping Core as part of the ACT study using previously established methods [29,30]. APOE e4 status is classified as 0 = absence of the e4 allele, 1 = 1 or 2 copies of the e4 allele. Data on Braak stages and Consortium to Establish a Registry for Alzheimer’s disease (CERAD) criteria for neuropathological diagnosis of Alzheimer’s disease were also collected. Braak criterion evaluates the density and distribution of neurofibrillary tangles and is categorized as 0 (none or normal), 1 (very mild), 2 (mild), 3 (moderate), 4 (moderately severe), 5 (severe), and 6 (very severe) [31,32]. CERAD criterion evaluates the highest density of neocortical neuritic plaques and is categorized as 0 (not identified), 1 (mild), 2 (moderate), and 3 (severe) [31,32].

### 2.5. Statistical Analysis

We conducted a cross-sectional design in which the associations of vasculopathies with microinfarcts were examined. For descriptive statistics, we compared characteristics of vasculopathies and microinfarcts, testing for statistical significance using Chi-square tests (all variables, including age, were categorical). Descriptive analysis was first performed for the overall cohort with and without cerebral microinfarcts (417 of the 842 participants had cerebral microinfarcts) and was then repeated among those with and without cortical (*n* = 301 of 842 had cortical) and subcortical (249 of 842 had subcortical) microinfarcts. To keep the sample size as large as possible, we did not exclude the patients with missing values. If a variable had a missing value, we converted the missing value into the category of “unknown”. Multivariable-adjusted logistic regression models were used to estimate odds ratios and 95% confidence intervals for the presence of cerebral microinfarcts associated with the two cerebral vasculopathies (arteriolosclerosis and CAA). Covariates included in the model were age, sex, late-life mean BP, antihypertensive medication use, *APOE* genotype, Braak, and CERAD stages. We then repeated the model to estimate adjusted OR (95%) for cortical and subcortical microinfarcts. Finally, we developed an ordered logistic regression model to estimate the associations of a cerebral vasculopathy (arteriolosclerosis, CAA, respectively) with the categorical number of microinfarcts, one and more than one, adjusting for the same covariates as above. We used absence or mild arteriolosclerosis as a reference group because only 8 participants had absent arteriolosclerosis (in comparison to absent CAA as a reference group). 

## 3. Results

### 3.1. Participant Characteristics 

Microinfarcts were present in 417 (49.5%) participants; 176 (42.2%) had one, and 241 (57.8%) had multiple. Cortical and sub-cortical microinfarcts were present in 301 (72.2%) and 249 (59.7%) participants, while 133 (31.9%) had both. CAA was present in 320 (38%) participants, arteriolosclerosis in 708 (84.1%), and 284 (34%) had both (Table 1).

### 3.2. The Associations between Covariates and Vasculopathies and Presence of Microinfarcts

The prevalence of microinfarct was significantly higher with older age, 54.7% in those aged ≥ 90 years, 38.8% in those 80–89 years, and 6% below 80. Females were not significantly more likely to have a microinfarct than males. They had higher last mean systolic (≥140 mmHg: 32.5% vs. 42.4%) and diastolic (≥90 mmHg: 3.1% vs. 7.7%) blood pressures and were more significantly likely to be taking antihypertensives (67.5% vs. 72.7%). There was minimal difference between those with or without APOE e4 (28.5% vs. 25.4%). Compared to participants without microinfarcts, those with a microinfarction had a higher prevalence of arteriolosclerosis (81.4% vs. 86.8%) but not of CAA (36.5% vs. 39.1%) (Table 1).

### 3.3. The Associations between Covariates and Vasculopathies and Location of Microinfarcts

The prevalence of microinfarct in both cortical and subcortical regions, respectively, were significantly higher with older age, 56.5% and 52.6% in those aged ≥ 90 years, 38.2% and 41.8% in those 80–89 years, and 5.3% and 5.6% below 80. Females were not significantly more likely to have both cortical and subcortical microinfarcts than males. They had higher last mean systolic (≥140 mmHg: 43.9% vs. 33.8% cortical, 46.2% vs. 33.7% subcortical) and diastolic (≥90 mmHg: 8.6% vs. 3.5 cortical, 7.6% vs. 4.4% subcortical) blood pressures and were more significantly likely be taking antihypertensives (72.8% vs. 68.6% cortical, 76.3% vs. 67.5% subcortical). There was minimal difference between those with or without APOE e4 (26.2% vs. 27.4% cortical, 26.1% vs. 27.3% subcortical). Participants with cortical microinfarcts had a higher prevalence of CAA (42.9% vs. 35.3%) but not arteriolosclerosis (80.1% vs. 82.4%). Those with subcortical microinfarcts had a higher prevalence of arteriolosclerosis (85.1% vs. 83.6%) but not of CAA (36.1% vs. 38.8%) (Table 1). 

### 3.4. Associations of Severity of Arteriolosclerosis with Presence, Number, and Location of Cerebral Microinfarcts 

No or mild arteriosclerosis was present in 58 (13.9%) and 128 (30.1%) participants with and without cerebral microinfarcts, respectively. Moderate arteriolosclerosis was present in 183 (43.9%) and 170 (40.0%) participants with and without cerebral microinfarcts (adjusted OR when compared with no or mild arteriolosclerosis, 2.16; 95% CI, 1.46–3.18; Table 2). Severe arteriolosclerosis was present in 124 (29.7%) and 53 (12.5%) participants with and without cerebral microinfarcts (adjusted OR when compared with no or mild arteriolosclerosis, 4.63; 95% CI, 2.90–7.40; Table 2). Moderate and severe arteriolosclerosis had similar associations with a number of microinfarcts (Table 2). Both moderate and severe arteriolosclerosis had significant associations with cortical microinfarcts, with adjusted Ors (95% Cis) of 1.86 (1.21–2.86) and 3.79 (2.35–6.12; Table 2). Respective adjusted Ors (95% Cis) for subcortical microinfarcts were 2.23 (1.38–3.61) and 4.04 (2.39–6.82; Table 2).

### 3.5. Associations of Severity of CAA with Presence, Number, and Severity of Cerebral Microinfarcts

No CAA was present in 254 (60.9%) and 268 (63.1%) participants with and without cerebral microinfarcts, respectively. Mild, moderate, and severe CAA was present in 75 (18.0%), 73 (17.5%), and 15 (3.6%) participants with cerebral microinfarcts, and 77 (18.1%), 70 (16.5%), and 10 (2.4%) of participants without cerebral microinfarcts, respectively (Table 1). When compared with no CAA, adjusted Ors (95% Cis) for cerebral microinfarcts associated with mild, moderate, and severe CAA were 0.93 (0.64–1.36), 1.02 (0.68–1.54), and 1.40 (0.60–3.29), respectively; (Table 2). Similar associations were observed with a number of microinfarcts (Table 2). When compared with no CAA, adjusted Ors (95% Cis) for cortical microinfarcts associated with mild, moderate, and severe CAA were 1.05 (0.71–1.56), 1.50 (0.99–2.27), and 1.69 (0.73–3.91), respectively; (Table 2). Respective adjusted Ors (95% Cis) for subcortical microinfarcts were 0.84 (0.55–1.28), 0.72 (0.46–1.14), and 0.92 (0.37–2.28); (Table 2).

## 4. Discussion

The findings from the current study demonstrate that cerebral microinfarcts were common in older adults, and their presence, number, and location were associated with a higher prevalence of arteriolosclerosis but not with CAA. We also observed that when adjusted for potential confounders, including age, sex, last systolic blood pressure, last diastolic blood pressure, APOE e4, Braak, and CERAD, the presence of moderate and severe arteriolosclerosis was associated with significantly higher odds of cerebral microinfarcts. Furthermore, this association was graded in that the higher severity of arteriolosclerosis was associated with higher odds of microinfarcts and that this association was similar for cortical and subcortical microinfarcts. Furthermore, we observed incrementally higher odds of microinfarcts in the presence, number, and cortical regions with higher severity of CAA. These findings suggest that the underlying pathophysiology of cerebral vasculopathies has a complex relationship with cerebral microinfarcts, a better understanding of which is necessary to develop appropriate interventions to lower the vasculopathy-associated risk of dementia. 

The Rush Memory and Aging Project and the Religious Orders Study, a large brain autopsy study (*n* = 1066) comparable to ours (*n* = 842), reported 18% significantly higher odds of having cerebral microinfarcts associated with the presence of severe arteriolosclerosis [7]. This is numerically lower than the odds of microinfarcts observed in our study. While underlying causes of these differences may lie in the differences in study populations and/or methodologies, it is also possible that there are differences in the underlying pathophysiology. The complexity is further compounded by the observation that in the Rush study, arteriolosclerosis had no association with cortical microinfarcts, while in our study, arteriolosclerosis had similar strong associations with both cortical and subcortical microinfarcts. While understanding the underlying causes of these differences is beyond the scope of the current study, these observations provide new information about potential heterogeneity in the complex and poorly understood relationship between arteriolosclerosis and cerebral microinfarcts. Although our study did not examine the association between atherosclerosis and microinfarcts, the Rush study reported a similar magnitude of the associations between atherosclerosis and arteriolosclerosis with microinfarcts [7]. This is interesting because atherosclerosis and arteriolosclerosis have different underlying pathophysiologic mechanisms, the former being atheromatous and the latter being non-atheromatous [33]. However, because both may coexist and contribute to microinfarct formation, it is difficult to estimate the true contribution of arteriolosclerosis [34,35].

The Rush Memory and Aging Project and the Religious Orders Study also provide useful background information for the interpretation of CAA and microinfarct observed in our study. A study based on the Rush Memory and Aging Project and the Religious Orders Study reported that higher severity levels of arteriolosclerosis increased the risk of one or multiple microinfarcts by about 20% and that amyloid angiopathy increased the risk by 13% [7]. In that study [7], the presence of CAA was associated with 13% higher odds of cerebral microinfarcts. In our study, the association between CAA and microinfarcts was numerically stronger (104% higher), but it was not significant, likely due to inadequate power. Similarly, there were differences in the magnitude of the associations between arteriolosclerosis and microinfarcts. However, like the Rush study, we also observed that CAA had no association with subcortical microinfarcts and that CAA was associated with 42% and 69% higher odds of having cortical microinfarcts. Although neither association was significant, likely for the same reason of inadequate power, the numerically stronger associations with more severe CAA suggest a dose-response in the relationship, which may provide additional insights into this relationship. 

Arteriolosclerosis and CAA are two possible major causes of cerebral microinfarcts, which in turn have long been known to be associated with neurodegenerative changes, leading to cognitive impairment and dementia, especially in late-life older adults. Small vessel diseases such as arteriolosclerosis could lead to cerebral hypoperfusion, fibrinoid necrosis, and degeneration of vascular myocytes, resulting in cerebral microinfarcts. In consequence, cerebral microinfarcts in both cortical and subcortical brain regions can lead to cognitive impairment and dementia by causing cumulative, brain-wide disruptions to neural connectivity, glial dysfunction, and neuroinflammation [15,36,37]. The relationship between these vasculopathies and cerebral microinfarcts has been variously reported by smaller autopsy studies [38,39]. Findings from the current study, taken together with the Rush study with a similar sample size, suggest that the relationship between vasculopathies and cerebral microinfarct is complex and yet to be fully understood. Considering the role of cerebral microinfarcts in the causation of dementia [40], it is important to better understand the pathophysiology of cerebral vasculopathies so that targeted interventions can be developed to lower the risk of dementia. People with Alzheimer’s disease diagnosed were found to have a high degree of vessel pathology (e.g., arteriolosclerosis, CAA), and the vessel pathology increases the risk of developing clinical dementia even in people with a low burden of AD pathology [24,37].

Despite this study being based on a relatively large autopsy sample from a longitudinal community-based cohort, we acknowledge our study’s limitations. We looked at only two vasculopathies such as CAA and arteriolosclerosis in relation to microinfarct genesis. There might be other factors influencing microinfarct formation. Microinfarcts were calculated from the four cortical and four subcortical brain regions. In addition, CAA was collected only in the brain leptomeninges and occipital cortex. Moreover, we could not assess the relationships between vasculopathies and microinfarcts in each individual region because the four brain regions were lumped into cortical and subcortical. All these may underestimate the total microinfarct and CAA burden in other regions. The autopsy-based microinfarcts data limit our inference on causality. It is possible that microinfarcts preceded CAA and arteriolosclerosis. The ACT participants with over 90% non-Hispanic white urban-dwelling enrolled in one of the largest managed care systems may limit generalizability to other population settings. In addition, the autopsy participants are not a random sample of the broader ACT sample. 

## 5. Conclusions

The findings of the current study demonstrate that cerebral microinfarcts were common in older adults. The presence of moderate and severe arteriolosclerosis had a significant association with the presence, number, and location (cortical and subcortical) of cerebral microinfarcts when potential confounders were adjusted. In addition, the association was graded in that higher severity in arteriolosclerosis had higher odds of microinfarcts. However, the association with CAA was weak and non-significant. These findings underscore the need for additional studies to better understand the complex relationship between additional different vasculopathies (e.g., vasculitis, lacunar infarcts) and cerebral microinfarcts so that interventions can be developed for primary prevention of vasculopathies, microinfarcts, and cognitive impairment. In addition, future studies can focus on whether incremental relationships are present in vasculopathies and cognition, such as in microinfarcts.

## Figures and Tables

**Table 1:** Characteristics of the Participants (*n* = 842).

Variables N/Mean (%/STD)	Microinfarcts Presence			Cortical Microinfarcts			Subcortical Microinfarcts		
	No (*n* = 425)	Yes (*n* = 417)	*p*	No (*n* = 541)	Yes (*n* = 301)	*p*	No (*n* = 593)	Yes (*n* = 249)	*p*
Age at death									
<80	68	27	<0.0001	79	16	<0.0001	81	14	<0.01
	(16.0)	(6.5)		(14.6)	(5.3)		(13.7)	(5.6)	
80–89	173	162		220	115		231	104	
	(40.7)	(38.8)		(40.7)	(38.2)		(39.0)	(41.8)	
90+	184	228		242	170		281	131	
	(43.3)	(54.7)		(44.7)	(56.5)		(47.4)	(52.6)	
Sex									
Male	185	171	0.46	231	125	0.74	248	98	0.27
	(43.5)	(41.0)		(42.7)	(41.5)		(43.5)	(39.4)	
Female	240	246		310	176		335	151	
	(56.5)	(59.0)		(57.3)	(58.5)		(56.5)	(60.6)	
APOE e4									
No	293	294	0.33	337	210	0.70	410	177	0.79
	(68.9)	(70.5)		(69.7)	(69.8)		(69.1)	(71.1)	
Yes	121	106		148	79		162	65	
	(28.5)	(25.4)		(27.4)	(26.2)		(27.3)	(26.1)	
Unknown	11	17		16	12		21	7	
	(2.6)	(4.1)		(3.0)	(4.0)		(3.5)	(2.8)	
Braak									
0	16	9	0.11	19	6	0.11	21	4	0.16
	(3.8)	(2.2)		(3.5)	(2.0)		(3.5)	(1.6)	
I–II	114	90		144	60		145	59	
	(26.8)	(21.6)		(26.6)	(19.9)		(24.5)	(23.7)	
III–IV	152	148		190	110		218	82	
	(35.8)	(35.5)		(35.1)	(36.5)		(36.8)	(32.9)	
V–VI	141	169		186	124		206	104	
	(33.2)	(40.5)		(34.4)	(41.2)		(34.7)	(41.8)	
Unknown	2	1		2	1		3	0	
	(0.5)	(0.2)		(0.4)	(0.3)		(0.5)	(0.0)	
CERAD									
Absent	105	86	0.15	130	61	0.11	143	48	0.41
	(24.7)	(20.6)		(24.0)	(20.3)		(24.1)	(19.3)	
Sparse	113	95		141	67		146	62	
	(26.6)	(22.8)		(26.1)	(22.3)		(24.6)	(24.9)	
Moderate	94	110		118	86		143	61	
	(22.1)	(26.4)		(21.8)	(28.6)		(24.1)	(24.5)	
Severe	113	126		152	87		161	78	
	(26.6)	(30.2)		(28.1)	(28.9)		(27.2)	(31.3)	
Antihypertensive Medication use									
No	138	112	0.08	169	81	0.39	193	57	<0.01
	(32.5)	(26.9)		(31.2)	(26.9)		(32.5)	(22.9)	
Yes	287	303		371	219		400	190	
	(67.5)	(72.7)		(68.6)	(72.8)		(67.5)	(76.3)	
Unknown	0	2		1	1		0	2	
	(0)	(0.5)		(0.2)	(0.3)		(0)	(0.8)	
Mean systolic blood pressure	132	136	<0.001	133	136	0.03	132	139	<0.0001
	(21.5)	(22.5)		(21.6)	(22.9)		(21.2)	(23.5)	
≥140 mmHg	138	177		183	132		200	115	
	(32.5)	(42.4)		(33.8)	(43.9)		(33.7)	(46.2)	
<140 mmHg	285	238	0.01	355	168	<0.01	390	133	<0.01
	(67.1)	(57.1)		(65.6)	(55.8)		(65.8)	(53.4)	
Unknown	2	2		3	1		3	1	
	(0.5)	(0.5)		(0.6)	(0.3)		(0.5)	(0.4)	
Mean diastolic blood pressure	69	71	0.02	70	71	0.14	70	72	<0.01
	(10.6)	(11.9)		(10.7)	(12.3)		(11.0)	(11.8)	
≥90 mmHg	13	32		19	26		26	19	
	(3.1)	(7.7)		(3.5)	(8.6)		(4.4)	(7.6)	
<90 mmHg	410	383	0.02	519	274	<0.01	564	229	0.16
	(96.5)	(91.8)		(95.9)	(91.0)		(95.1)	(92.0)	
Unknown	2	2		3	1		3	1	
	(0.5)	(0.5)		(0.6)	(0.3)		(0.5)	(0.4)	
Cerebral amyloid angiopathy									
Absent	268	254	0.71	350	172	0.07	363	159	0.82
	(63.1)	(60.9)		(64.7)	(57.1)		(61.2)	(63.9)	
Mild	77	75		97	55		108	44	
	(18.1)	(18.0)		(17.9)	(18.3)		(18.2)	(17.7)	
Moderate	70	73		81	62		105	38	
	(16.5)	(17.5)		(15.0)	(20.6)		(17.7)	(15.3)	
Severe	10	15		13	12		17	8	
	(2.4)	(3.6)		(2.4)	(4.0)		(2.9)	(3.2)	
Arteriolosclerosis									
Absent	5	3	<0.0001	7	1	<0.0001	5	3	<0.0001
	(1.2)	(0.7)		(1.3)	(0.3)		(0.8)	(1.2)	
Mild	123	55		139	39		153	25	
	(28.9)	(13.2)		(25.7)	(13.0)		(25.8)	(10.0)	
Moderate	170	183		226	127		245	108	
	(40.0)	(43.9)		(41.8)	(42.2)		(41.3)	(43.4)	
Severe	53	124		81	96		98	79	
	(12.5)	(29.7)		(15.0)	(31.9)		(16.5)	(31.7)	
Unknown	74	52		88	38		92	34	
	(17.4)	(12.5)		(16.3)	(12.6)		(15.5)	(13.7)	
Dementia (yes)	116	215	<0.01	223	158	<0.01	243	138	<0.01
	(39.1)	(51.6)		(41.2)	(52.5)		(41.0)	(55.4)	

**Table 2:** Association of cerebral amyloid angiopathy and arteriolosclerosis with microinfarcts presence.

Variables	Adjusted Odds Ratio (95% CI)			
	Microinfarcts (Presence)	Microinfarcts (Numbers)	Microinfarcts (Cortical)	Microinfarcts (Subcortical)
Arteriolosclerosis ^1^				
Moderate	2.16 (1.46–3.18) *	2.25 (1.54–3.30) *	1.86 (1.21–2.86) **	2.23 (1.38–3.61) **
Severe	4.63 (2.90–7.40) *	4.91 (3.18–7.60) *	3.79 (2.35–6.12) *	4.04 (2.39–6.82) *
Unknown	1.61 (0.98–2.62)	2.20 (1.38–3.52) **	1.69 (0.99–2.89)	2.13 (1.19–3.82) ***
Cerebral amyloid angiopathy ^2^				
Mild	0.93 (0.64–1.36)	0.95 (0.66–1.35)	1.05 (0.71–1.56)	0.84 (0.55–1.28)
Moderate	1.02 (0.68–1.54)	1.04 (0.71–1.52)	1.50 (0.99–2.27)	0.72 (0.46–1.14)
Severe	1.40 (0.60–3.29)	2.05 (0.94–4.45)	1.69 (0.73–3.91)	0.92 (0.37–2.28)

Note: * *p* ≤ 0.0001, ** *p* ≤ 0.001, *** *p* ≤ 0.01. Adjusted for last systolic blood pressure, last diastolic blood pressure, APOE e4 genotype, age, sex, Braak, CERAD, and antihypertensive medication use. ^1^ Reference: absence or mild arteriolosclerosis. ^2^ Reference: absence of cerebral amyloid angiopathy.

## Data Availability

The data presented in this study are available on request from the ACT. For more information you can visit: https://actagingstudy.org/ (accessed on 4 May 2022).
